# Predictors of ileal pouch–anal anastomosis failure in ulcerative colitis: a European tertiary referral center experience

**DOI:** 10.1007/s10151-026-03395-5

**Published:** 2026-07-17

**Authors:** T. Violante, G. Calini, S. Cardelli, M. Novelli, F. Rizzello, W. R. Perry, M. Rottoli

**Affiliations:** 1 Surgery of the Alimentary Tract, IRCCS Azienda Ospedaliero-Universitaria Di Bologna, Bologna, Italy; 2 Department of Medical and Surgical Sciences, Alma Mater Studiorum - University of Bologna, Via Massarenti 9, 40138 Bologna, Italy; 3 Division of Colon and Rectal Surgery, Mayo Clinic, Rochester, MN USA; 4 Department of Statistical Sciences, Alma Mater Studiorum - University of Bologna, Bologna, Italy; 5 Inflammatory Bowel Disease Unit, IRCCS Azienda Ospedaliero-Universitaria Di Bologna, University of Bologna, Bologna, Italy

**Keywords:** Ulcerative colitis, Proctocolectomy, restorative, Biological therapy, Nomograms, Treatment failure

## Abstract

**Background:**

Total proctocolectomy with ileal pouch–anal anastomosis serves as the standard surgical treatment for medically refractory ulcerative colitis, yet long-term pouch failure remains a significant clinical challenge. This study aimed to identify independent predictors of pouch failure and to develop a practical clinical nomogram for individualized risk assessment.

**Methods:**

A retrospective cohort study was conducted utilizing a prospectively maintained database at a European tertiary referral center, analyzing procedures performed between December 2005 and June 2025. The study included 851 adult patients with a confirmed histologic diagnosis of ulcerative colitis who underwent restorative surgery. The primary outcome was pouch failure, defined as permanent pouch excision or indefinite fecal diversion. Independent predictors were identified using penalized logistic regression modeling to build a predictive risk tool.

**Results:**

Over a median follow-up of 37.0 months for the entire cohort, pouch failure occurred in 4.7% of patients (*n* = 40). Among patients who experienced pouch loss, the median time to failure was 29.4 months. The majority of these failures manifested after 12 months and were primarily driven by severe fistulae and chronic leaks. Preoperative biologic therapy emerged as the sole independent predictor of pouch failure. Handsewn anastomoses and non-standard pouch configurations also demonstrated strong associations with failure. A five-variable clinical nomogram was constructed to provide individualized failure probabilities.

**Conclusion:**

While overall pouch failure rates remain low in specialized centers, exposure to preoperative biologic therapy significantly elevates this risk. The newly developed nomogram provides a valuable, evidence-based tool for tailored surgical counseling and personalized patient management.

**Supplementary Information:**

The online version contains supplementary material available at https://doi.org/10.1007/s10151-026-03395-5.

## Introduction

Total proctocolectomy with ileal pouch–anal anastomosis (IPAA) is the standard restorative procedure for patients with medically refractory or complicated ulcerative colitis, allowing preservation of transanal defecation and avoidance of a permanent ileostomy [1]. Since its introduction in 1978, refinements in patient selection, perioperative optimization, and surgical technique have led to excellent long-term functional outcomes in the majority of patients [2]. Nevertheless, a proportion of pouches ultimately fail, requiring permanent fecal diversion or pouch excision, with profound impact on quality of life and healthcare utilization [3]. Reported rates of pouch failure after IPAA vary widely, ranging from approximately 2% to 15% in population-based series and meta-analyses, depending on the length of follow-up and case mix [4]. Pouch failure is most commonly driven by pelvic sepsis, chronic or refractory pouchitis, fistulizing disease, anastomotic complications, and Crohn’s-like disease of the pouch [5, 6]. Several clinical and technical risk factors have been proposed, including male sex, high body mass index, advanced age, extraintestinal manifestations, and perioperative immunosuppression, but data are heterogeneous and often derived from mixed inflammatory bowel disease cohorts or pre-biologic eras [7–10].

In the biologic era, increasing use of advanced medical therapies, evolving indications, and more complex surgical candidates may have altered both the incidence and pattern of pouch failure. Recent studies suggest that biologic exposure, pelvic sepsis, chronic pouchitis, low institutional volume, and technical variables such as anastomotic technique may independently influence long-term pouch survival, yet robust, contemporaneous data focused exclusively on ulcerative colitis remain limited [11–13]. Moreover, the relative contribution of modifiable perioperative and surgical factors, particularly in high-risk patients, has not been fully quantified, and few models provide clinically usable risk stratification at the time of IPAA [14].​

Our institution is a high-volume European tertiary referral center for inflammatory bowel disease surgery and IPAA, with a large prospectively maintained database of patients with ulcerative colitis. In this context, we aimed to identify independent clinical and surgical predictors of pouch failure in a homogeneous cohort of patients with ulcerative colitis treated between 2005 and 2025, and to develop a pragmatic clinical risk nomogram to support individualized counselling, operative planning, and postoperative surveillance.

## Methods

This retrospective cohort study was conducted using a prospectively maintained institutional database of patients who underwent IPAA between December 2005 and June 2025 at the IRCCS Azienda Ospedaliero-Universitaria di Bologna, a tertiary referral center for inflammatory bowel disease. The primary objective was to identify independent clinical and surgical predictors of long-term pouch failure. This study received IRB approval and adhered to STROBE guidelines.

From an initial database of IPAA procedures performed during the study period, stringent inclusion and exclusion criteria were applied to establish a homogeneous study cohort. Inclusion was restricted to adult patients with a confirmed histologic diagnosis of ulcerative colitis. Patients with familial adenomatous polyposis, Crohn’s disease, or indeterminate colitis were excluded. To ensure accurate time-to-event analysis, patients with missing or zero months of postoperative follow-up were excluded. Furthermore, to eliminate the confounding influence of the learning curve and technical differences associated with newly adopted technologies, patients undergoing robotic or robotic-converted approaches were excluded.

Patient demographics, preoperative clinical status, and intraoperative variables were extracted from the electronic medical record. Baseline variables included age at the time of IPAA, sex, and body mass index (BMI). Smoking history was dichotomized into active smokers versus all others (never, former, or unknown), and the American Society of Anesthesiologists (ASA) physical status classification was grouped into ASA I–II (no or mild systemic disease) versus ASA III–IV (severe systemic disease).

Disease and surgical variables included the use of biologic therapy at the time of IPAA (defined as the administration of any biologic agent within 12 weeks prior to the IPAA procedure), surgical approach (open, laparoscopic, or laparoscopic converted to open), anastomosis technique (stapled versus handsewn), and pouch configuration. Pouch anatomy was categorized as a standard J-pouch versus non-standard configurations (S-pouch or W-pouch). Handsewn anastomoses were strictly utilized for specific clinical indications, such as a short mesentery or the need for a mucosectomy due to dysplasia risk. Non-standard pouch configurations (S- or W-pouches) were employed exclusively as rescue techniques when severe mesenteric tension prevented a standard J-pouch from reaching the pelvis. Consequently, these approaches inherently reflect technically complex and high-risk cases. For patients receiving preoperative biologic therapy, the procedural staging was additionally recorded and classified as two-stage (proctocolectomy, IPAA and diverting loop ileostomy, followed by ileostomy closure) versus three-stage (subtotal colectomy with end ileostomy, followed by proctectomy with IPAA and diverting loop ileostomy, and finally ileostomy closure).

The primary outcome of the study was pouch failure, defined as permanent pouch excision or indefinite fecal diversion with any type of ileostomy. Secondary outcomes included the etiology of pouch failure and the timing of failure, categorized as early (< 12 months) or late (≥ 12 months) after IPAA. Time-to-event was calculated in months from the date of the index IPAA surgery to the date of pouch failure or the date of last clinical follow-up.

### Statistical analysis

Continuous variables were reported as medians with interquartile ranges (IQR) and compared between the failure and non-failure groups using the Mann–Whitney* U* test, as the data did not assume a normal distribution. Categorical variables were reported as frequencies with percentages and compared using Pearson’s chi-square test or Fisher’s exact test where appropriate. Time-to-event analysis was performed using the Kaplan–Meier method to estimate long-term pouch survival. Standard logistic regression was utilized for the primary multivariable analysis specifically to facilitate the construction of a simplified, integer-based clinical nomogram for preoperative bedside use.

To identify independent predictors of pouch failure while mitigating the risks of model overfitting and multicollinearity, a least absolute shrinkage and selection operator (LASSO) logistic regression was employed. The LASSO algorithm applies an L1 penalty that shrinks the coefficients of weak or uninformative variables toward zero, effectively removing them from the model. The optimal penalization parameter was selected using fivefold cross-validation. Continuous variables were standardized prior to LASSO modeling.

To obtain interpretable clinical estimates, a post-LASSO multivariable logistic regression was subsequently performed. Only the non-zero variables selected by the LASSO algorithm were entered into this unpenalized multivariable model, yielding adjusted odds ratios (OR), 95% confidence intervals (CI), and *p* values.

Finally, to facilitate clinical translation, a predictive clinical risk nomogram was constructed by assigning proportional integer points to the standardized coefficients of the retained LASSO predictors.

To enhance clinical utility and facilitate clinical application, a streamlined predictive nomogram was constructed focusing on the five variables demonstrating the strongest associations with pouch failure in the post-LASSO multivariable analysis: preoperative biologic use, non-J-pouch configuration, handsewn anastomosis, laparoscopic-to-open conversion, and ASA class III–IV status. The nomogram was developed by assigning proportional integer points (0–100) to the standardized coefficients of these five key predictors. The “total points” axis represents the linear predictor, which was then mapped non-linearly to the predicted probability of pouch failure to account for the logistic nature of the underlying model.

All statistical analyses were performed using StataNow 19 SE (StataCorp, College Station, TX, USA). A two-sided *p* value of < 0.05 was considered statistically significant.

## Results

A total of 851 patients met the inclusion criteria (Table 1). The cohort had a median age of 44.0 years (IQR 32.0–55.0) at the time of surgery and was predominantly male (59.7%). The median BMI was 23.4 kg/m^2^ (IQR 20.8–26.0). Preoperative biologic therapy was utilized in 18.7% (*n* = 159) of patients. Among the 159 biologic-exposed patients, the primary agents utilized were infliximab (*n* = 132), adalimumab (*n* = 15), vedolizumab (*n* = 10), and other biologics (*n* = 11). Within this biologic-exposed subgroup, 103 patients underwent a two-stage procedure, while 56 underwent a three-stage approach. Most surgeries were performed via an open approach (80.1%), with 17.4% completed laparoscopically and 2.1% requiring conversion from laparoscopy to open. Anastomoses were predominantly stapled (90.0%), and nearly all patients received a standard J-pouch configuration (99.6%). An era-based sensitivity analysis (pre-2015 vs. post-2015) revealed significant practice shifts over the study period. Pre-2015, only approximately 5% of patients received preoperative biologics, and minimally invasive approaches were rare. Post-2015, biologic utilization increased to nearly 30%, and laparoscopy became the dominant surgical approach (> 60%).

**Table 1:** Patient demographics and cohort description

Baseline characteristic	Total cohort (*N* = 851)	Successful pouch (*N* = 811)	Pouch failure (*N* = 40)	*p* value
Median age at IPAA, median (IQR)	44.0 (32.0—55.0)	44.0 (32.0—55.0)	44.0 (30.5—51.3)	0.683
Sex (male)	508 (59.7%)	483 (59.6%)	25 (62.5%)	0.844
Median BMI, median (IQR)	23.4 (20.8—26.0)	23.4 (20.8—26.0)	23.7 (20.8—26.1)	0.919
Smoking status				0.638
Active smoker	177 (20.8%)	167 (20.6%)	10 (25.0%)	
Other (never/ex/unknown)	674 (79.2%)	644 (79.4%)	30 (75.0%)	
ASA score				0.201
ASA I–II (healthy/mild)	729 (85.7%)	698 (86.1%)	31 (77.5%)	
ASA III–IV (severe)	122 (14.3%)	113 (13.9%)	9 (22.5%)	
Preoperative biologics				0.006
No	673 (81.3%)	650 (82.1%)	23 (57.5%)	
Yes	159 (18.7%)	145 (17.9%)	14 (35.0%)	
Surgical approach				0.324
Open	682 (80.1%)	650 (80.1%)	32 (80.0%)	
Laparoscopic	148 (17.4%)	143 (17.6%)	5 (12.5%)	
Laparoscopic converted to open	18 (2.1%)	16 (2.0%)	2 (5.0%)	
IPAA anastomosis type				0.012
Stapled	754 (90.0%)	724 (90.8%)	30 (75.0%)	
Handsewn	84 (10.0%)	75 (9.2%)	9 (22.5%)	
Pouch configuration				< 0.001
J-pouch	848 (99.6%)	810 (99.9%)	38 (95.0%)	
Non-J-pouch (S or W)	3 (0.4%)	1 (0.1%)	2 (5.0%)	

This table describes the baseline population and highlights the initial unadjusted comparisons between patients who retained their pouch and those who lost it

Data are presented as median (IQR) or* n* (%)

*IPAA* ileal pouch–anal anastomosis,* IQR* interquartile range,* BMI* body mass index,* ASA* American Society of Anesthesiologists,* Lap* laparoscopic

The median follow-up for the overall cohort, including censored patients, was 37.0 months. Pouch failure occurred in 40 patients, representing a crude failure rate of 4.7%. Within the failure group, the median time from surgery to pouch excision or permanent diversion was 29.4 months (IQR 15.6–71.1 months). On univariate analysis, patients who experienced pouch failure were significantly more likely to have received preoperative biologic therapy (35.0% vs. 17.9%, *p* = 0.006), to have undergone a handsewn anastomosis (22.5% vs. 9.2%, *p* = 0.012), or to have a non-standard pouch configuration (5.0% vs. 0.1%, *p* < 0.001). The most common etiologies leading to pouch failure were severe pouch fistulae (*n* = 16, 40.0%), chronic anastomotic leaks (*n* = 11, 27.5%), and refractory pouchitis (*n* = 9, 22.5%). Less frequent causes included cuffitis (*n* = 8) and stricture or stenosis (*n* = 5), while true progression to Crohn’s disease of the pouch was identified in only one patient.

**Fig. 1:**
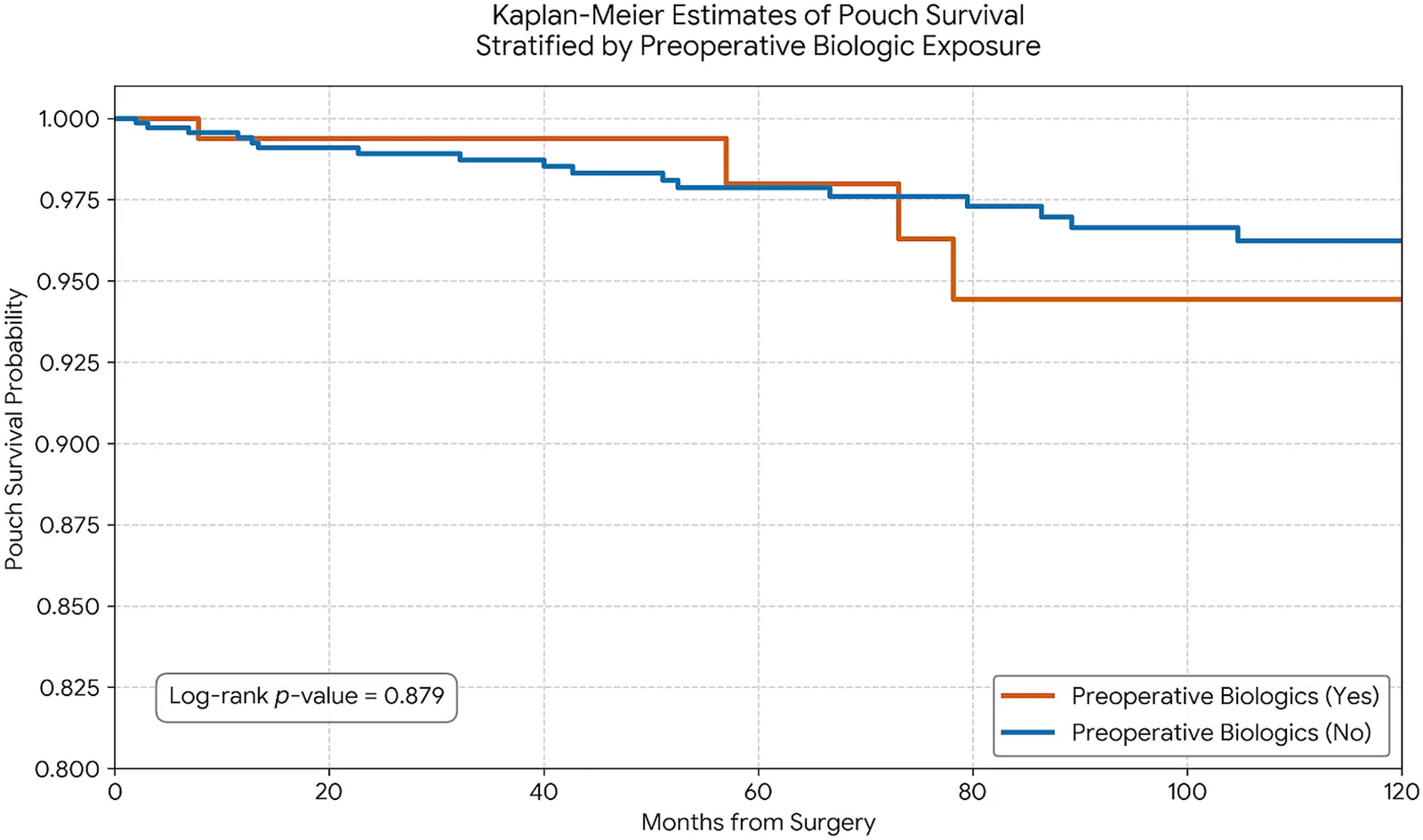
Kaplan–Meier estimates of pouch survival stratified by preoperative biologic exposure. Overall pouch survival probability is shown up to 10 years (120 months) post-surgery for patients with ulcerative colitis undergoing ileal pouch–anal anastomosis (IPAA). A log-rank test revealed no statistically significant difference in long-term time-to-failure between the two groups (log-rank *p* = 0.879)

Timing of failure was skewed toward late complications, with 33 patients (82.5%) experiencing pouch loss ≥ 12 months postoperatively, compared with 7 patients (17.5%) with early failure (< 12 months). Early pouch loss was significantly associated with intraoperative surgical difficulty, as 28.6% of early failures occurred in patients requiring conversion from laparoscopic to open surgery, compared with none among late failures (*p* = 0.007). Conversely, late pouch failures were predominantly related to medical intractability, with preoperative biologic use present in 43.3% of late failures compared with 14.3% of early failures. According to Kaplan–Meier survival analysis, the estimated 5-year pouch survival rate was 97.9%, and the 10-year survival rate was 96.0% (Fig. 1).

To identify independent risk factors while accounting for multicollinearity, a LASSO logistic regression model was employed (Table 2). The LASSO algorithm shrank non-predictive variables to zero and retained preoperative biologic therapy, handsewn anastomosis, laparoscopic-to-open conversion, active smoking, ASA III–IV status, non-J-pouch configuration, open surgical approach, and younger age as predictors of pouch failure.

**Table 2:** LASSO logistic regression predictors for pouch failure

Clinical feature	Standardized LASSO coefficient (*β*)
Preoperative biologics (vs. no biologics)	+ 0.316
Hand-sewn anastomosis (vs. stapled)	+ 0.236
Laparoscopic converted to open (vs. successful minimally invasive)	+ 0.189
Active smoker (vs. never/ex-smoker)	+ 0.158
ASA score III–IV (vs. ASA I–II)	+ 0.141
Non-J-pouch (vs. standard J-pouch)	+ 0.123
Open surgical approach (vs. laparoscopic)	+ 0.077
Age at IPAA (per year increase)	− 0.058

*LASSO* least absolute shrinkage and selection operator, *ASA* American Society of Anesthesiologists, *IPAA* ileal pouch–anal anastomosis, *Lap* laparoscopic

These selected variables were then evaluated in a post-LASSO unpenalized multivariable logistic regression to calculate adjusted risk (Table 3). In this model, preoperative biologic therapy independently more than doubled the odds of pouch failure (OR 2.28, 95% CI 1.11–4.69, *p* = 0.025). Handsewn anastomosis (OR 2.23, 95% CI 0.92–5.39, *p* = 0.075) and non-J-pouch configuration (OR 12.12, 95% CI 0.93–157.96, *p* = 0.057) were associated with markedly elevated odds ratios but bordered on statistical significance, likely as a result of the small number of patients undergoing these specific techniques. Other variables, including laparoscopic conversion, ASA III–IV status, age, active smoking, and open approach, showed weaker and non-significant associations in the multivariable model.

**Table 3:** Post-LASSO multivariable logistic regression predictors for pouch failure

Clinical feature	Adjusted odds ratio	95% confidence interval	*p* value
Preoperative biologics (vs. no biologics)	2.28	1.11–4.69	0.025
Non-J-pouch (vs. standard J-pouch)	12.12	0.93–157.96	0.057
Handsewn anastomosis (vs. stapled)	2.23	0.92–5.39	0.075
Lap converted to open (vs. minimally invasive)	3.34	0.60–18.51	0.167
ASA score III–IV (vs. ASA I–II)	1.80	0.78–4.17	0.170
Age at IPAA (per year increase)	0.99	0.97–1.02	0.488
Active smoker (vs. never/ex-smoker)	1.31	0.58–2.94	0.512
Open surgical approach (vs. minimally invasive)	1.20	0.47–3.06	0.706

Variables selected by the initial LASSO model were fit into an unpenalized multivariable regression to calculate unadjusted odds ratios and *p* values. Non-selected variables were excluded from this step

*LASSO* least absolute shrinkage and selection operator, *CI* confidence interval, *IPAA* ileal pouch–anal anastomosis, *ASA* American Society of Anesthesiologists, *Lap* laparoscopic

**Fig. 2:**
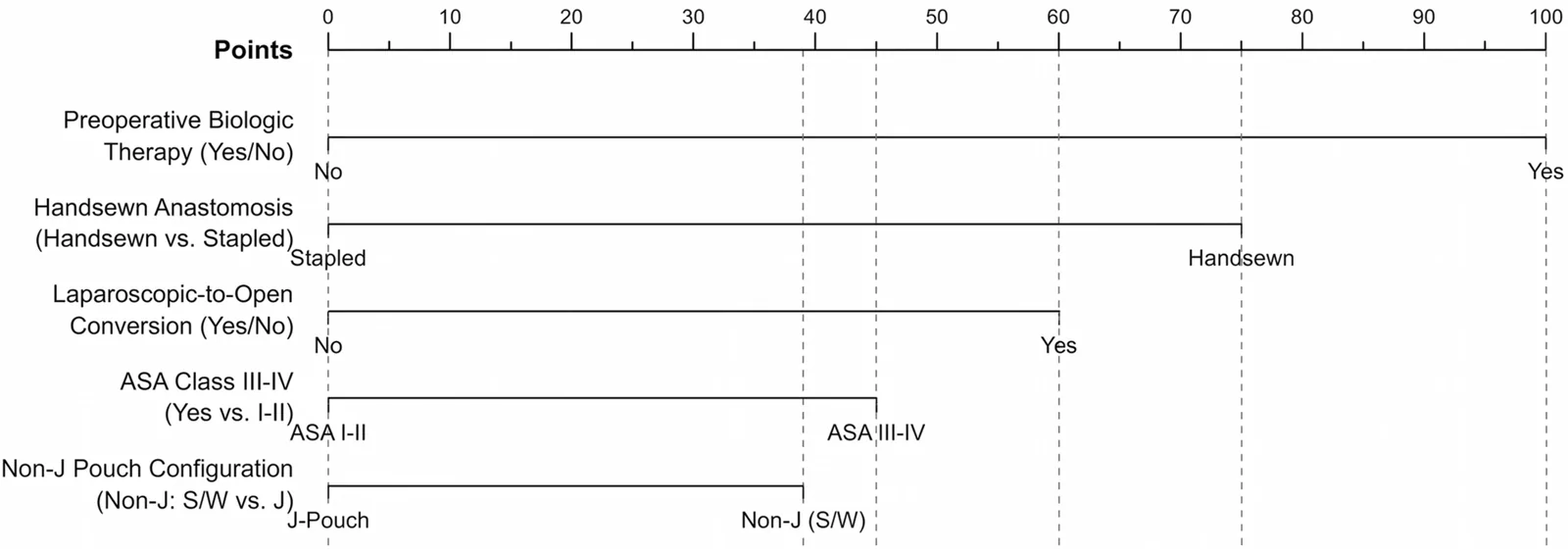
Predictive clinical risk nomogram for long-term pouch failure. The tool is derived from a post-LASSO multivariable logistic regression model of 851 patients. To use the nomogram: (1) locate the patient’s status on each of the five variable axes; (2) draw a vertical line upward to the “points” axis to determine the score for each; (3) sum the points to calculate the “totalpoints”; and (4) locate the total points on the bottom axis and draw a vertical line downward to identify the individualized predicted probability of long-term failure

A visual, individualized risk estimation tool (nomogram, Fig. 2) was developed on the basis of the post-LASSO analysis. To maximize clinical utility, a refined, simplified model was also constructed, focusing on the five variables demonstrating the strongest associations with long-term IPAA failure. These key predictors included preoperative biologic use (the sole independent predictor, adjusted OR 2.28, *p* = 0.025), non-J-pouch configuration, handsewn anastomosis, laparoscopic-to-open conversion, and ASA class III–IV status. By assigning proportional integer points based on the beta coefficients of each retained predictor, the tool allows clinicians to calculate a cumulative “total points” score for any given patient, which maps non-linearly to an estimated probability of long-term pouch failure ranging from < 1% to > 30%.

## Discussion

In this large single-center cohort of 851 patients with ulcerative colitis undergoing IPAA, the overall pouch failure rate of 4.7% after a median follow-up of 37 months lies at the lower end of contemporary estimates, which typically range between 5% and 15% at medium- to long-term follow-up. Most failures occurred beyond 12 months and were chiefly related to fistulizing complications, chronic anastomotic leakage, and refractory pouchitis or cuffitis, supporting the concept that pouch loss usually reflects the cumulative effect of chronic sepsis and inflammation rather than isolated early technical failure. Using a LASSO-based selection strategy followed by post-LASSO multivariable logistic regression, we identified preoperative biologic therapy as the only independent predictor of pouch failure, with handsewn anastomosis and non-J-pouch configuration showing strong but imprecise associations.

Our institutional failure rate compares favorably with population-based and multicenter series, where permanent pouch loss is reported in approximately 7–12% of patients and continues to increase beyond 10–15 years of follow-up [15, 16]. A large systematic review including almost 23,000 patients found a pooled failure rate of 7.7%, rising to over 10% in cohorts with at least 10 years of follow-up, underlining the importance of time horizon when comparing outcomes across studies [4]. Similarly, long-term series have described pouch success rates of 85–90% at 10–15 years, with quality of life generally acceptable but reduced in the presence of pouchitis, perianal inflammation, or high stool frequency [17]. Within this context, our data suggests that, in a high-volume tertiary referral center with homogeneous ulcerative colitis case mix and standardized technique, durable pouch survival is achievable in the vast majority of patients in the biologic era [18]. The predominance of late failures and the central role of fistulae and chronic leaks in our cohort align with prior analyses identifying pouch fistula, often reflecting an occult or chronic leak, as a major determinant of long-term pouch loss [19, 20].

Preoperative biologic therapy more than doubled the odds of pouch failure in our adjusted model, in keeping with emerging multicenter data implicating biologic exposure, pelvic sepsis, chronic pouchitis, and low institutional volume as key drivers of adverse long-term outcomes [21]. Biologic treatment likely acts as a composite marker of aggressive, refractory disease and may also exert direct effects on anastomotic healing, microbial composition, and mucosal immunity, thereby predisposing to early sepsis and subsequent chronic pouch inflammation [22].

Handsewn IPAA and non-J-pouch configurations were associated with markedly elevated odds of pouch failure, though neither reached conventional statistical significance in the post-LASSO multivariable model (*p* = 0.075 and *p* = 0.057, respectively). Given this, these findings should be considered exploratory and hypothesis-generating. Rather than implicating the surgical techniques themselves, the elevated risk likely reflects selection bias; these approaches are inherently reserved for technically demanding, high-risk cases characterized by severe distal rectal disease, dysplasia, or restricted mesenteric reach. Nevertheless, our observations align with prior studies linking handsewn anastomoses and complex pouch designs (S- or W-pouches) to higher rates of pelvic sepsis, strictures, and long-term dysfunction [23, 24]. Ultimately, these findings reinforce current clinical guidelines that favor the stapled J-pouch as the standard of care, reserving handsewn anastomoses and alternative configurations for highly selected indications [25].

Other variables selected by the LASSO algorithm, including conversion from laparoscopy to open surgery, ASA class III–IV, active smoking, younger age, and open approach, did not remain independently associated with failure in the post-LASSO model. Conversion, however, retained a relatively high point allocation in the risk nomogram, suggesting that it may function as a surrogate marker of operative complexity and severe pelvic inflammation rather than a directly modifiable risk factor [26, 27]. The lack of a strong independent effect of ASA status or operative access route is consistent with recent large series indicating that, once technical and disease-related factors are controlled for, comorbidity burden and laparoscopic versus open approach exert limited influence on long-term pouch survival.

An important contribution of this study is the translation of penalized regression into a pragmatic bedside tool for individual risk stratification. LASSO regression is particularly suited to settings with numerous, potentially collinear candidate variables and relatively few events, as it reduces overfitting by shrinking weak coefficients towards zero while preserving clinically relevant predictors. Building on this framework, we derived a simple nomogram that integrates preoperative biologic use, handsewn anastomosis, laparoscopic conversion, active smoking, ASA III–IV status, and non-J-pouch configuration into a cumulative point score to estimate the probability of pouch failure at the time of IPAA planning.

It is important to note that recognized postoperative drivers of pouch failure, such as pelvic sepsis and clinically evident anastomotic leaks, were intentionally excluded from the predictive model. The nomogram was designed strictly as a preoperative and intraoperative risk-stratification tool to forecast risk before postoperative complications occur, thereby helping surgeons anticipate and potentially mitigate these exact downstream events.

In practice, a young non-smoking patient without biologic exposure, with ASA I–II status, scheduled for stapled J-pouch IPAA, and uncomplicated laparoscopic or open access would accrue a low-risk score and a very low predicted probability of pouch loss. Conversely, an actively smoking, comorbid patient who has received biologics and requires conversion to open surgery with a handsewn non-J-pouch would accumulate a high score, indicating a substantially increased risk and justifying more intensive counselling, meticulous leak-prevention strategies, and closer long-term surveillance. By offering a nuanced, multifactorial estimate rather than a binary “high-risk/low-risk” classification, the nomogram can facilitate shared decision-making and help tailor both operative strategy and follow-up intensity.

These findings have several implications for multidisciplinary management of ulcerative colitis candidates for restorative proctocolectomy. First, the strong association between biologic exposure and pouch failure supports early surgical referral and consideration of staged colectomy in patients with refractory disease who have failed multiple biologics, especially when additional risk factors such as smoking or high ASA class are present. Second, the data emphasize the importance of standardized high-quality technique, specifically, routine stapled J-pouch IPAA performed by experienced colorectal surgeons, as a modifiable lever to reduce chronic leaks, fistulae, and functional impairment. Third, the predominance of late, sepsis- and inflammation-driven failures highlights the need for structured, long-term surveillance protocols with a low threshold for imaging and pouch endoscopy in patients with symptoms suggestive of subclinical leaks or refractory pouchitis, coupled with aggressive medical and interventional management to prevent irreversible damage.

Several limitations must be acknowledged. First, the use of standard logistic regression for a time-to-event outcome is methodologically suboptimal compared to Cox proportional hazards or Fine–Gray models, as it does not account for censoring or variable follow-up time. This approach was chosen explicitly to derive a pragmatic, integer-based clinical scoring system for the nomogram, but it remains a methodological limitation. Furthermore, the retrospective design introduces the potential for residual confounding and information bias, and although the overall cohort was large, the absolute number of failures was modest, leading to wide confidence intervals and some instability in effect estimates. As a single high-volume European tertiary center, our results may not be directly generalizable to lower-volume institutions with different case mixes, technical expertise, or perioperative pathways. Moreover, although LASSO-based modelling reduces overfitting, our nomogram has not yet undergone external validation, and its calibration and discrimination in independent populations remain to be determined. Finally, we focused on structural pouch failure and did not include functional outcomes or patient-reported measures, which are increasingly recognized as critical endpoints in restorative proctocolectomy. Furthermore, we cannot entirely rule out residual confounding by disease severity. Because granular clinical severity indices (e.g., Mayo scores) and cumulative steroid doses were not universally recorded across the entire 20-year retrospective span, “preoperative biologic therapy” likely acts as a composite proxy for a refractory, aggressive disease phenotype rather than an isolated pharmacological risk factor.

Future studies should externally validate and, if needed, recalibrate this nomogram in multicenter ulcerative colitis IPAA cohorts with differing volumes and surgical strategies. Prospective work should also test whether embedding the risk score into multidisciplinary pathways, guiding staging, anastomotic technique, and surveillance intensity, reduces pouch failure and improves quality of life. In parallel, mechanistic research on biologic exposure, the microbiome, and host immunity may identify adjunctive therapies to limit chronic pouch inflammation and pelvic sepsis. Ultimately, integrating validated prediction tools with individualized medical and surgical strategies may further improve long-term pouch survival and function.

## Conclusion

In this large single-center IPAA cohort, pouch failure was uncommon (4.7% at approx. 5 years) and predominantly late, mainly related to fistulae, chronic anastomotic leaks, and refractory pouchitis/cuffitis. Preoperative biologic therapy independently increased the risk of pouch loss, while handsewn anastomosis and non-J-pouch configuration showed strong but imprecise associations, underscoring the need for careful patient selection and standardized pouch construction. Our nomogram may assist in individualized risk stratification and in tailoring counselling, surgical strategy, and follow-up in patients with ulcerative colitis considered for IPAA in the biologic era.

## Supplementary Information

Below is the link to the electronic supplementary material.

Supplementary file1 (DOCX 33 KB)

## Data Availability

No datasets were generated or analyzed during the current study.

## References

[1] Le Berre C, Honap S, Peyrin-Biroulet L (2023) Ulcerative colitis. Lancet 402(10401):571–584 10.1016/S0140-6736(23)00966-2 37573077

[2] Violante T, Ferrari D, Gomaa IA et al (2024) Evolution of laparoscopic ileal pouch-anal anastomosis: impact of enhanced recovery program, medication changes, and staged approaches on outcomes. J Gastrointest Surg 28(4):501–506 10.1016/j.gassur.2024.01.005 38583902

[3] Violante T, Behm KT, Shawki SF et al (2024) Robotic-assisted reoperative ileal pouch-anal anastomosis: robotic pouch excision and pouch revision. Tech Coloproctol 28(1):43 10.1007/s10151-024-02918-2 38561571

[4] Alsafi Z, Snell A, Segal JP (2022) Prevalence of “pouch failure” of the ileoanal pouch in ulcerative colitis: a systematic review and meta-analysis. Int J Colorectal Dis 37(2):357–364 10.1007/s00384-021-04067-6 PMC8803821 34825957

[5] Santiago P, Barnes EL, Raffals LE (2023) Classification and management of disorders of the J pouch. Am J Gastroenterol 118(11):1931–1939 10.14309/ajg.0000000000002348 37252759

[6] Maspero M, Lavryk O, Lipman J et al (2023) Redo ileal pouch-anal anastomosis for early versus late sepsis-related pouch failure. Surgery 174(4):801–807 10.1016/j.surg.2023.06.016 37543468

[7] Holubar SD, Russell T, Neel N et al (2026) Biologics before surgery are not associated with postoperative complications after surgery for inflammatory bowel disease: a national surgery quality improvement program inflammatory bowel disease collaborative causal inference analysis. Dis Colon Rectum. 10.1097/DCR.0000000000004077 PMC13036565 41492952

[8] Emile SH, Khan SM, Wexner SD (2021) A systematic review and meta-analysis of the outcome of ileal pouch anal anastomosis in patients with obesity. Surgery 170(6):1629–1636 10.1016/j.surg.2021.06.009 34226045

[9] McKenna NP, Mathis KL, Khasawneh MA et al (2017) Obese patients undergoing ileal pouch-anal anastomosis: short- and long-term surgical outcomes. Inflamm Bowel Dis 23(12):2142–2146 10.1097/MIB.0000000000001238 28922254

[10] Wu XR, Zhu H, Kiran RP, Remzi FH, Shen B (2013) Excessive weight gain is associated with an increased risk for pouch failure in patients with restorative proctocolectomy. Inflamm Bowel Dis 19(10):2173–2181 10.1097/MIB.0b013e31829bfc26 23899541

[11] Rencuzogullari A, Akyol C, Hamzaoglu I et al (2025) Predictors of pouch failure and quality of life following ileal pouch-anal anastomosis for ulcerative colitis: a retrospective multicenter study. ANZ J Surg 95(3):457–463 10.1111/ans.19336 39641425

[12] Chaouch MA, Hussain MI, Gouader A et al (2024) Stapled anastomosis versus hand-sewn anastomosis with mucosectomy for ileal pouch-anal anastomosis: a systematic review and meta-analysis of postoperative outcomes, functional outcomes, and oncological safety. Cancer Control 31:10732748241236338 10.1177/10732748241236338 PMC10898296 38410083

[13] Kotze PG, Avellaneda N, Moretti RAM et al (2024) Controversies in IPAA for ulcerative colitis: a systematic review of different anastomotic techniques. Dis Colon Rectum 67(S1):S26-S35 10.1097/DCR.0000000000003292 38710588

[14] Fazio VW, Tekkis PP, Remzi F et al (2003) Quantification of risk for pouch failure after ileal pouch anal anastomosis surgery. Ann Surg 238(4):605–614. 10.1097/01.sla.0000090940.39838.6a PMC1360119 14530732

[15] Mark-Christensen A, Erichsen R, Brandsborg S et al (2018) Pouch failures following ileal pouch-anal anastomosis for ulcerative colitis. Colorectal Dis 20(1):44–52 10.1111/codi.13802 28667683

[16] Violante T, Broccard SP, Novelli M et al (2025) Comparative analysis of robotic, laparoscopic, and open ileal pouch-anal anastomosis outcomes: retrospective cohort study. BJS Open. 10.1093/bjsopen/zraf084 PMC12312352 40742352

[17] Violante T, Ferrari D, McKenna NP et al (2025) Bowel function and quality of life after robotic and laparoscopic IPAA: a patient-reported outcomes study. J Robot Surg 19(1):360 10.1007/s11701-025-02488-x 40624405

[18] Morita S, Okabayashi K, Kitagawa Y et al (2026) textbook outcome after ileal pouch-anal anastomosis for ulcerative colitis: a nationwide multicenter study and its implications for long-term quality of life. Dis Colon Rectum. 10.1097/DCR.0000000000004157 41685680

[19] Bronze S, Kayal M, Estevinho MM et al (2025) Ileoanal pouch-related fistulas: a narrative review. Inflamm Bowel Dis 31(6):1716–1722 10.1093/ibd/izae221 39349403

[20] Tulchinsky H, Hawley PR, Nicholls J (2003) Long-term failure after restorative proctocolectomy for ulcerative colitis. Ann Surg 238(2):229–234 10.1097/01.sla.0000082121.84763.4c PMC1422693 12894016

[21] Ricardo AP, Kayal M, Plietz MC et al (2023) Predictors of pouch failure: a tertiary care inflammatory bowel disease centre experience. Colorectal Dis 25(7):1469–1478 10.1111/codi.16589 PMC12180589 37128185

[22] Lee KE, Sizemore JA, Kim G, Shen B, Sands BE (2024) Impact of biologics and small-molecule agents on postoperative complications in IBD: a systematic review. Dis Colon Rectum 67(S1):S11–S25 10.1097/DCR.0000000000003222 38294838

[23] Simillis C, Afxentiou T, Pellino G et al (2018) A systematic review and meta-analysis comparing adverse events and functional outcomes of different pouch designs after restorative proctocolectomy. Colorectal Dis 20(8):664–675 10.1111/codi.14104 29577558

[24] Reijntjes MA, Bocharewicz EK, Hompes R, Buskens CJ, Bemelman WA (2022) Incidence and causes of failure in various anatomical pouch designs 20 years after surgical primary ileal-pouch anal anastomosis construction. Int J Colorectal Dis 37(12):2491–2499 10.1007/s00384-022-04280-x PMC9741566 36357735

[25] Kirat HT, Remzi FH, Kiran RP, Fazio VW (2009) Comparison of outcomes after hand-sewn versus stapled ileal pouch-anal anastomosis in 3109 patients. Surgery 146(4):723–729. 10.1016/j.surg.2009.06.041 19789032

[26] Metzger DA, Mesiti A, Johnson J et al (2025) Pouch outcomes after minimally invasive vs. open proctectomy during IPAA reconstruction. Surg Endosc 39(4):2436–2442 10.1007/s00464-025-11574-y 39979620

[27] Lavryk OA, Stocchi L, Ashburn JH et al (2018) Case-matched comparison of long-term functional and quality of life outcomes following laparoscopic versus open ileal pouch-anal anastomosis. World J Surg 42(11):3746–3754 10.1007/s00268-018-4602-1 29785696

